# Supplementary material for: Novel Tetraploid Triticale (Einkorn Wheat × Rye)—A Source of Stem Rust Resistance
Source: Plants (Basel). 2023 Jan 7;12(2):278. doi: 10.3390/plants12020278 (PMC9865463; doi:10.3390/plants12020278)
Supplement: Supplementary file 1 [file plants-12-00278-s001.zip › plants-1919072-supplementary.pdf]

Supplementary Table S1. Statistics, analysis of variance followed by Tukey's Honest Significant Difference test (HSD) for infection test scores of sixty plants of S_4 generation; 30 plants of control genotypes: *T. aestivum* "Thatcher", (susceptible control) and *T. aestivum* Thatcher+*Sr57* (moderate resistant control).

**Summary values**		**1**	**2**	**3**
		**A^mA^mRR amphiloids (S_4 generation)**	***T. aestivum* Thatcher+ *Sr57***	***T. aestivum* "Thatcher"**
Number of samples		60	30	30
Mean		0.45	1.43	3.87
Range		0-1	1-2	3-4
Variance		0.124576	0.2254023	0.11954
Standard deviation		0.352954	0.504007	0.345746
Standard error		0.045566	0.092019	0.063124

**Analysis of variance (ANOVA)—treatment between groups**		
F-value		752.89
P-value		<0.0001
**Tukey's Highest Significant Difference (HSD) between any two sample means at designated level**		
1 vs2		P < 0.01
1vs 3		P < 0.01
2vs3		P < 0.01
**Significance level**	HSD [0.05]	0.22
	HSD [0.01]	0.28
